# Evaluation of the effects of digital play addiction on eating attitudes

**DOI:** 10.12669/pjms.342.14537

**Published:** 2018

**Authors:** Burcu Kayhan Tetik, Duygu Kayhan, Serap Sertkaya, Kamuran Bahar Sandikci

**Affiliations:** 1 Dr. Burcu Kayhan Tetik, Assistant Professor, Department of Family Medicine, Department of Family Medicine, İnönü University of Medical Faculty, Malatya, Turkey; 2 Duygu Kayhan, Psychological Counselor, Malatya Guidance Research Center, Malatya, Turkey. Malatya Guidance Research Center, Malatya, Turkey; 3 Dr. Serap Sertkaya, Department of Family Medicine, Department of Family Medicine, İnönü University of Medical Faculty, Malatya, Turkey; 4 Dr. Kamuran Bahar Sandikci, Karsiyaka Kamil Nalbant Family Health Center, Kocaeli, Turkey. Karsiyaka Kamil Nalbant Family Health Center, Kocaeli, Turkey

**Keywords:** Adolescent, Digital Games, Eating Habit

## Abstract

**Objective:**

To evaluate the impact of digital game addiction on the eating habits of adolescents aged between 9 and 15.

**Methods:**

This cross-sectional study was conducted as a survey of 972 middle school students. All data was evaluated with SPSS 22 software, and p<0.05 was accepted as statistically significant.

**Results:**

The mean age of the students was 12.0±1.29. It was found that male students spent more time playing digital games than the females and the more they played, the higher the risk level of their Eating Habit Index became, and the difference for both groups was statistically significant (p<0.001). It was also determined that students from the houses with easy access to internet tend to play digital games for longer periods of time (p<0.001).

**Conclusion:**

As a result, children at adolescence age need to be taught which games, how long and when to play and be able to limit them instead of getting banned from playing. We are of the opinion that subjects of digital game addiction and appropriate and efficient use of computers need to be included in the curriculum within the scope of Media Literacy and Information Technologies and Software lessons in order to raise the children’s awareness.

## INTRODUCTION

Playing activities play a very important role in the cognitive development of children and adolescents. Experts suggest that the daily digital gaming time should not exceed two hours. When this duration is exceeded, problems such as communication problems, antisocial personality traits, tendency to violence, lack of connection between virtual and real life, learning disabilities, early maturation of children, and decline in academic achievement can happen.^1^

With the correct usage, technology can prove to increase creativity, and can provide a more comfortable life, but it can also be harmful to a childs learning skills. Therefore families have the responsibility of limiting time spent on internet and computers.^2^ It has been stated that, particularly in children who are introduced to strategy games at younger ages, the ability of reasoning may be disrupted and it may create confusion in the inner world and can blunt conscience.^3^ To avoid such situations, parents need to examine the content and the original purpose of these games.

Adolescence is a period of great importance for adulthood. This is a risky period in which unhealthy eating behavior can be seen.^4^ During this period, eating habits can be influenced by many factors, including genetic factors, dislike of foods, digital game addiction, and psychological problems. The typical characteristics of unhealthy eating habits include, irregular meals, snack patterns between meals, routinely eating outside the home, and fast foods. These habits are often affected by many factors including family, peers, and the media.^5^ The World Health Organization (WHO) defines the 10-19 age group as adolescents and 15-24 age group as youth. According to WHO, 29.1% of the total population is under the age of 15, in Turkey.^5^ This period covers especially the secondary school period in Turkey.

It this study we aim to determine the frequency of digital game addiction in the secondary school age group and to investigate whether or not digital game addiction has an effect on eating habits.

## METHODS

This study was conducted as a cross-sectional descriptive study and ethical approval was obtained from the Board of Scientific Research and Publication of Inonu University with the Decision No. 2017 / 6-1. In order for the study to be carried out in secondary schools, written permission was obtained from the Provincial Directorate of National Education of Malatya Province with the number 61316475-44-E.4006094. The research was determined by a random sampling method in which three state schools and one private school among secondary schools in Battalgazi and Yesilyurt districts of Malatya were selected. In the sample size analysis with 95% confidence interval and 90% power, the minimum number of the sample was determined as 628.

A total of 1100 questionnaires were published which consisted of questions including the sociodemographic characteristics of the students and the Nutrition Habit Index Scale. Nutrition Habit Index Scale is a 5-point Likert scale consisting of a total of 6 questions, which were formed by Demirezen et al., in the adolescent student group, in 1999 and revised by them in 2005.^5^ The answers are scored as never = 0, rarely = 1, sometimes = 2, often = 3, and always = 4 points and the scores are classified as 0 points = no risk, 1-6 points = mild risk, 7-12 = moderate Risk, 13-18 points = high risk, and 19-24 points = very high risk. Due to absenteeism, only 90% of the students in the school were reached. The students who did not want to participate in the questionnaire on voluntary basis and those who did not want to answer some questions were excluded from the survey and the study was completed with a total of 972 students. The statistical analyses are performed by using IBM SPSS for Windows version 22.0 software. Arithmetic mean (X) ± standard deviation (SD) or median (min-max) values are used to define quantitative variables. Numerical variables are compared by using Mann Whitney U test for two independent groups and Kruskal-Wallis test for more than two groups and afterwards Conover binary comparison method is used. Discrete variables are defined by number (n) and percentage (%) and Pearson Chi-square test is used in the comparisons. In all tests, a value of p<0.05 is considered statistically significant.

## RESULTS

The median age was 12.0 ± 1.29 (min = 9, max = 15) and 522 (53.7%) of the students who participated in the study were male and 450 (46.3%) were female. Socio-demographic characteristics of the children are given in Table-I.

**Table-I:** Socio demographic characteristics of students.

	Number (n)	Percentage (%)
*Gender*		
Female	450	46.3
Male	522	53.7
Age	12.0	
BMI*	19.10	
*Grade*		
5^th^	295	30.3
6^th^ Grade	233	24.0
7^th^ Grade	167	17.2
8^th^ Grade	277	28.5
*Having Internet at Home*		
Yes	640	65.3
No	332	34.2
NHIS*Risk Levels		
Low Risk	225	23.1
Moderate Risk	527	54.2
High Risk	193	19.9
Very High Risk	27	2.8

* BMI: Body Mass Index,

NHIS: Nutrition Habit Index Scale.

The comparison of the students regarding gender revealed that girls spend an average of 48 minutes playing on the internet, while boys spend an average of 1.5 hours; which was found statistically significant (p<0.001).

A total of 640 students (65.8%) were reported to have internet access with one hour and 24 minutes average time spent playing digital games, whereas the students who do not have internet access at home spent an average of 42 minutes (p<0.001). With respect to the dietary habits they were practicing, it was identified that 23.1% of the students were at “low” risk level, 54.2% of them were at “moderate”, %19.9 of the participants were at “high” and %2.8 were at “very high” risk level. (Table 2). (P<0.001). More male students were found to be included in “high risk group” regarding the dietary habits, in comparison to female students (24.5%); and it is also ascertained that “low risk” dietary habit was more prevalent among the students within 9-12 age group (29.5%) (p=0.000). In addition, more 8th grade students were noted to fall into “high risk” and “very high risk” groups than those of other grades. (p=0.000). On the other hand, the use of internet and the BMI scores were found to present no significant differentiation with regard to dietary habits. (p>0.05).

**Table-II:** Distribution of nutrition habits index risk assessment of the students (n=972).

NHIS			

Low Risk (n/%)	Moderate Risk (n/%)	High Risk (n/%)	Very High Risk (n/%)
225(23.1)	527(54.2)	193(19.9)	27(2.8)

When risky nutritional levels of NHIS were compared regarding availability of internet at home, we found that most of the children were in the moderate risk group with a percentage of 54.2% in both groups; there was no statistical difference (p=0.380).

We further found that the students in the low and moderate risk group according to the NHIS scores spent an average of 30 minutes playing digital games on the internet (p>0.05). The students in the high risk group according to the NHIS scores spent an average of 1.5 hours and the students in the very high risk group spent 2.5 hours in playing digital games and it was found to be statistically significant (p<0.05). There was no statistically significant difference between the durations students spent on the internet in relation to BMI (p=0.925). The average BMI of the low risk group was 18.47 ± 3.3, the average BMI of the moderate risk group was 19.35 ± 8.7, the average BMI of the high risk group was 19.2 ± 3.8, and the average BMI of the very high risk group was 18.52 ± 2.8, and these differences were not statistically significant (p=0.34). There was no statistically significant difference between the NHIS risk levels in terms of the occupation of the parents of the students (p>0.05).

**Table-III:** The NHIS risk group’s values calculation according to the demographic variables.

	NHIS				Test

	Low Risk	Moderate Risk	High Risk	Very High Risk	
*Gender*					
Female	118 (26.2)	258 (57.3)	65(14.4)	9(2.1)	X^2=^19.104 p=0.000*
Male	107(20.5)	269(51.5)	128(24.5)	18(3.5)	
*Age*					
9-12 age	158(29.5)	282(52.7)	86(16.1)	9(1.7)	X^2=^35.888 p=0.000*
13-15 age	66(15.2)	244(56.1)	107(24.6)	18(4.1)	
*Grade*					
5^th^ Grade	101(34.2)	154(52.2)	37(12.5)	3(1.1)	X^2=^49.055 p=0.000*
6^th^ Grade	55(23.6)	122(52.4)	49(21.0)	7(3.0)	
7^th^ Grade	30(18.0)	98(58.7)	35(21.0)	4(2.3)	
8^th^ Grade	39(14.1)	153(55.2)	72(26.0)	13(4.7)	
*Having Internet at Home*					
Yes	141(22.0)	347(54.2)	131(20.5)	21(3.3)	X^2=^3.074 p=0.380
No	84(25.3)	180(54.2)	62(18.7)	6(1.8)	
*BMI*					
Weak	117(25.9)	239(52.9)	83(18.4)	13(2.8)	X^2=^13.668 p=0.135
Normal weight	84(21.7)	212(54.8)	77(19.9)	14(3.6)	
Over weight	5(10.9)	26(56.5)	15(32.6)	-	
Obese	-	5(71.4)	2(28.6)	-	

* p<0.001.

## DISCUSSION

Digital games are seen as pastime activities for children and adolescents as well as adults. However, the attraction to digital games can sometimes lead to a level of dependence for children and adolescents who experience difficulties in limiting themselves in almost everything.

A review of the literature reveals that boys play more digital games than girls^6^. However, there are studies suggesting the opposite.^7^ In our study, we found that boys play more digital games than girls. This divergence among the studies may be due to the fact that studies are conducted in different age groups and in communities with different cultural characteristics.

In the previous studies, it has been determined that 22.6% to 85% of children who have home computers use their computers for playing games.^8^ In our study this rate was 65.8%. This high rate is particularly likely to be due to the fact that secondary school students may still be fond of games when compared to high school students and high school students may have reached a certain satisfaction over time.

It has been stated that both internet and digital game dependency increase with the increase of the number of hours spent on the internet.^9^ Adolescents experience a feeling of happiness while playing a game and with increasing duration of playing digital game this feeling intensifies. Therefore they want to prolong the time spent in the game to maintain this feeling further. At the same time, the situations they enjoy before lose importance and they feel deprived and become aggressive when they are restricted from play, and when they begin playing again, they become calm and happy. They experience conflict with their family and teachers about the game playing process and school success declines and eating habits worsens.

In the study of Kiran et al., they found that the students spent 2-3 hours at the computer.^10^ Mannikko et al. found that students aged 16-18 played digital games an average of 2.5 hours a day.^11^ In our study, this rate was one hours and 6 minutes, and the students who have internet access at home spent an average of one hours and 24 minutes playing digital games, and the students who don’t have internet access spent an average of 42 minutes. On the contrary to the literature, the lower duration of playing digital games, in our study can be attributed to the fact that the children participated in our study were younger and consequently the authority of the family may be more effective on the child. Although playing digital games seems to contribute to healthy life, abreaction, and relaxation, especially in adolescents, in the literature, it has been pointed out that, if not controlled, it can lead to serious health problems including nutrition disorders.^12^ Kim and Chum investigated the relationship between internet addiction and nutrition habits and they found that the nutritional content of the meals of the children with internet addiction is poorer. In a study measuring the effects of digital gaming on nutrition habits in university students, it was found that as the duration of playing digital games increases, negative eating habits increases, minds constantly occupied and primary needs such as nutrition lost importance. They also observed that main meals are skipped and snacking was preferred.^7^ In another study conducted on students, in 2004, it was stated that as the duration of playing internet games increased, nutrition habits deteriorated and children became depressed.^13^ Tao et al. in their study conducted in Chinese schools, concluded that digital game addiction disrupted eating habits and caused obesity.^14^ Similarly, Smahel et al. reported that children with internet addiction encountered problems including nutrition disorders.^15^ Similarly, we found that there is a positive relationship between the increase in the duration of digital gaming and risky eating habits.

However, in the literature, there are studies reporting that children’s eating habits and health related self-management behaviors are improved, and even BMI is reduced by using digital games.^16^,^17^ There are also contradictory studies reporting that digital games increase BMI in children.^18^ Mannickko et al^11^ in their study found no relationship between digital game addiction and BMI. In our study, there was no relation between nutrition habit and children’s BMI. Due to these differences in the literature, we believe that it is appropriate to conduct more extensive studies.

Our study is an important because of the sample size and because the questionnaire is filled by face to face interview technique with each child. Since questions that children cannot understand were explained immediately, the questionnaire is filled completely. However, since it is planned as a cross-sectional study, whether any awareness is created in children cannot be observed. In addition, since the questions were not asked to the families of the children, they may not have been objective about how they assessed themselves. The aim of our study was to show the relationship between digital game addiction and symptoms of nutrition disorders, but there is need for studies with wider participation.

## CONCLUSION

It is necessary to teach children the timing, duration, and types of digital games as well as how to limit themselves, instead of forbidding them to play. In addition, it is also necessary to play games with children or adolescents whenever it is possible or talk with them, in order to evaluate whether they are aware of difference between the virtual and real worlds. We also suggest that the media literacy, information technologies and software courses in the curriculum should include information on digital game dependency and appropriate and efficient use of computers. We hope that, particularly in our country where young population is larger and there is a lack of supervision and protective measures in the face of rapid development of digital technologies, our study will contribute to overcome digital game addiction.

### Authors’ Contribution

BKT conceived, designed and did statistical analysis & editing of manuscript.

KBS, did manuscript writing.

DK did data collection. SS did statistical analysis.

BKT takes the responsibility and is accountable for all aspects of the work in ensuring that questions related to the accuracy or integrity of any part of the work are appropriately investigated and resolved.

All authors have revised the manuscript.
